# Predictors of willingness to eat wild mushrooms: extended theory of reasoned action

**DOI:** 10.3389/fnut.2025.1553392

**Published:** 2025-05-20

**Authors:** Fangmin Gong, Jingwen Zhuang, Han Liu, Zhenyi Li, Yuhan Jia, Juan Du, Xincheng Huang, Si Chen

**Affiliations:** ^1^College of Literature and Journalism Communication, Jishou University, Jishou, China; ^2^Department of Communication and Culture, Royal Roads University, Victoria, BC, Canada; ^3^Office of the Party and Administration, Hebei Academy of Fine Arts, Shijiazhuang, China; ^4^School of Journalism and Communication, South China University of Technology, Guangzhou, Guangdong, China; ^5^China National Center for Food Safety Risk Assessment, Beijing, China

**Keywords:** attitude, eating wild mushrooms, perceived benefit, self-efficacy, subjective norms

## Abstract

**Background:**

The accidental consumption of wild poisonous mushrooms has emerged as a primary source of poisoning incidents globally. It is imperative to comprehend the dietary habits of individuals consuming wild mushrooms to address this issue effectively.

**Methods:**

In this investigation, an extended version of the theory of reasoned action was employed, incorporating perceived benefit and food-related self-efficacy as novel predictive variables.

**Results:**

A total of 793 Chinese residents participated in the study, which revealed that subjective norms (β = 0. 219, *t* = 5.314), attitudes (β = 0.426, *t* = 8.237), self-efficacy (β = 0.144, *t* = 5.905), and perceived benefit (β = 0.177, *t* = 4.586) significantly influenced the participants' intentions.

**Conclusion:**

The extended theory of reasoned action framework has proven to be a valuable instrument for understanding individuals' inclinations toward selecting food-related risks. These factors should be considered in governmental initiatives aimed at enhancing food safety.

## 1 Introduction

The consumption of wild mushrooms is deeply popular in China, having long been regarded as a mountain delicacy by residents of many regions. It is estimated that over 1,000 species of poisonous mushrooms exist globally, with China alone accounting for at least 500 species (1). However, accidental ingestion of poisonous wild mushrooms has become the primary cause of poisoning incidents worldwide (2, 3). In some Asian countries, mushroom poisoning poses a severe public health threat, with China reporting the highest mortality rates (4). Poisonous mushroom poisoning in China is characterized by a high case fatality rate and substantial regional prevalence, far exceeding the global average (5). Among food-borne poisoning cases in China, mushroom poisoning incidents are the most frequent, accounting for 44.35% of total cases in 2022—a staggering increase of 88.35% compared to the previous year (6). Studies in multiple regions of China have identified poisonous mushroom poisoning as the primary cause of localized food-borne disease outbreaks and fatalities (7–9). Effective governmental management and control measures are urgently needed to mitigate the risks associated with wild mushroom consumption (10).

Due to the continuous increase in cases of accidental ingestion of poisonous wild mushrooms, many countries have implemented measures to address this issue. For example, numerous European countries have established legal frameworks to ensure the safe trade of wild mushrooms (11). The American Association of Poison Control Centers (AAPC) requires individuals to submit their wild mushroom specimens to local experts and provides telephone support for pharmacists, doctors, and nurses through poison centers (12). In China, high-risk regions such as Yunnan, Hubei, Jiangsu, Hunan, Sichuan, and Anhui have activated early warning systems during peak seasons. These systems employ a variety of communication channels, including radio broadcasts, bulletin boards, posters, distribution of educational materials, and public meetings. In remote mountainous areas, risk prevention efforts are further enhanced through radio broadcasts, permanent displays of warning posters, and community gatherings. Radio broadcasts leverage their accessibility and immediacy to rapidly disseminate risk warnings and identification knowledge during peak seasons or in specific regions, thereby raising public vigilance. Bulletin boards and posters use text-and-image combinations to provide long-term educational displays in densely populated areas, reinforcing awareness of food safety. Public meetings facilitate direct interaction between government agencies and health departments, enabling precise transmission of risk-related information and addressing public concerns, thereby fostering a sense of responsibility and engagement among participants. These measures integrate the strengths of diverse media channels to comprehensively elevate public awareness and promote the adoption of the “Four No's” principle: not picking, not eating, not selling, and not purchasing wild mushrooms (13, 14). Despite localized successes in reducing poisoning incidents, the overall risk of wild mushroom consumption remains inadequately managed (10). Poisonous mushroom poisoning has thus become a major cause of poisoning globally (15).

The striking similarity in macroscopic characteristics between edible and poisonous mushrooms makes them easily confused in mixed wild habitats (16). According to the latest annual report from the American Association of Poison Control Centers (AAPCC), 1,119 mushroom exposure cases were reported in 2022, with only 63.37% successfully identified (17). Furthermore, mushroom identification requires specialized expertise, which often exceeds the capacity of healthcare professionals (18). Folk verbal descriptions of mushrooms are subjective as their morphology varies with seasons, growth stages, local habitats, and environmental conditions (19). The incidence of mushroom poisoning varies significantly worldwide due to local traditions, lifestyles, nutritional factors, climate, and the presence of wild mushrooms (4). For untrained individuals, relying on methods such as Internet searches to distinguish mushrooms is insufficient, and misidentification could potentially compromise patient care (20). In addition, the vast majority of mushroom poisoning cases lack antidotes (21). Consequently, the only definitive way to prevent poisoning is to refrain from picking, consuming, selling, or purchasing wild mushrooms.

Herbert Simon proposed that decision-makers in risk behaviors are social individuals with bounded rationality, as their choices are constrained by environmental factors and limited cognitive capacities, often leading to behaviors that are less than fully rational or even irrational (22). Therefore, understanding the influencing factors of risky behaviors, such as the consumption of wild mushrooms, is essential to address cognitive biases and risk preferences in individuals, thereby promoting health-protective behaviors. This understanding also serves as a prerequisite for government-led interventions. However, research aimed at improving food safety behavior theories remains limited, and the lack of theoretical guidance continues to hinder advancements in food safety education (23, 24), resulting in ineffective intervention programs (3, 25).

This study addresses the dual challenges of theoretical and practical limitations by exploring key factors influencing the willingness to eat wild mushrooms. The theoretical framework and model integrate the theory of reasoned action (TRA) and a risk-benefit perspective. The TRA was selected because it has been successfully applied in numerous studies on consumer food choice behaviors (26, 27). However, the traditional TRA model has two critical limitations: first, its “attitude” variable implicitly encompasses a comprehensive evaluation of behavioral outcomes but fails to distinguish the independent roles of “risk” and “benefit.” Second, the TRA does not account for individuals' confidence in their ability to manage risks, which self-efficacy theory can explain—specifically, why individuals with high self-efficacy may overlook warnings due to overconfidence.

Given that perceived benefit and food self-efficacy are critical predictors in food safety research (28, 29), and prior studies have not integrated the TRA with these constructs, this research integrates the TRA, perceived benefit, and self-efficacy into a unified model. This integration provides empirical evidence to explain consumers' willingness to eat wild mushrooms. The study aims to validate this extended theoretical framework using structural equation modeling (PLS-SEM), clarifying the predictive pathways of subjective norms, attitude, perceived benefit, and self-efficacy on consumption intentions. The findings will offer empirical evidence for public health policies, enabling the design of targeted intervention strategies. This research holds significant public health value for reducing global mushroom poisoning incidents.

## 2 Literature review and research hypotheses

### 2.1 Theory of reasoned action

Many studies have utilized behavioral science theories and models to better understand food handlers' behaviors (30–32). Among these, the theory of reasoned action (TRA) is the most widely applied, having been used to study food-handling practices among consumers in Australia, Malaysia, the United States, and China (33). Proposed by Ajzen and Fishbein, TRA primarily analyzes how and why attitudes influence behavior, explaining the causal relationships between attitudes, subjective norms, and behavioral intentions (34). Existing literature demonstrates that TRA is highly effective in predicting and explaining food safety behaviors. For instance, Hosseini et al. (35) and Janani and Annapoorni (36) separately demonstrated the effectiveness of TRA-based interventions in promoting breakfast consumption and intentions to purchase organic foods. In addition, Xiaopeng et al. constructed an extended TRA model to investigate consumers' willingness to purchase green agricultural products (37).

The theory of reasoned action (TRA) directly predicts individual behavior through behavioral intention, which is jointly influenced by two primary factors: attitude and subjective norms (38). Attitude refers to an individual or organization's emotional evaluation of a behavior, encompassing both positive and negative orientations toward it. For instance, Harris et al. (39) found that customers' attitudes significantly influence their willingness to patronize restaurants with reported foodborne illness cases. Rodrigues et al. (40) revealed that food handlers' intentions to implement food safety behaviors are directly shaped by their attitudes toward such practices. Rezaei et al. (41) noted that positive attitudes toward food safety behaviors enhance Iranian farmers' willingness to engage in on-farm food safety practices. Petrovici and Paliwoda (42) further demonstrated that attitudes and habits significantly impact food consumption behaviors.

In China, the consumption of wild mushrooms is deeply rooted in cultural traditions. Wild mushrooms are revered as “mountain delicacies” and celebrated as “pure natural foods” (43). In many regions, they are not only a staple of daily diets but also play a central role in festivals and social gatherings (44). Their perceived high nutritional value has made wild mushrooms a cherished food choice among consumers (45). In addition, Chinese culinary culture surrounding wild mushrooms is profoundly influenced by regional traditions and generationally accumulated knowledge. Rural residents, in particular, have developed unique systems of mushroom identification and consumption through familial and communal knowledge transmission (46). This cultural legacy fosters a positive attitude toward wild mushrooms among Chinese consumers, positioning them as a food of high value. Consequently, in China's cultural context, attitude plays a critical role in understanding the decision-making processes of consumers regarding wild mushroom consumption.

Subjective norms refer to the perceived social pressure individuals or organizations feel when deciding whether to engage in a specific behavior (47). Long considered a powerful determinant of behavior, subjective norms have been extensively studied in behavioral research (48). In dietary choices, scholars have noted that individuals may conform to others' food-related norms to guide their eating behaviors (49). For instance, studies show that participants consume less of food if they perceive it as the standard of an unpopular social group (50, 51). Such social-environmental pressures can make it harder for people to maintain healthy diets, as they may lack the motivation to deviate from the choices of their social circles, even if those choices involve unhealthy but palatable options (52). Unhealthy social norms can provide reasons for behavioral change, potentially encouraging even health-conscious individuals to adopt less healthy eating habits to align with the majority (48). Notably, this influence often operates unconsciously as consumers may not recognize the impact of subjective norms (53). Recent research further highlights the significant role of subjective norms in shaping sustainable food choices (54). However, scholars have also noted that subjective norms are not always decisive in influencing behavior (55).

China emphasizes collective, where profound societal influence on individual decision-making, where family, community, and broader social norms exert deeply ingrained impacts (56). In the context of wild mushroom consumption, an individual's attitude is shaped not only by personal preferences but also by the opinions of family members and the community. For instance, individuals are more likely to exhibit a higher willingness to consume wild mushrooms if their family members or peers hold positive attitudes toward them (33). This powerful social influence aligns closely with the subjective norms construct in the theory of reasoned action (TRA), suggesting that subjective norms may be a critical predictor of behavioral intentions in China's cultural context.

Based on the above discussion, this study proposes the following hypotheses:

H1: Attitude positively affects the willingness to eat wild mushrooms.
H2: Subjective norms positively affect the willingness to eat wild mushrooms.

### 2.2 Perceived benefit, self-efficacy

Consumer perceptions of a food's benefits are often emotionally driven (57). Emotion can be defined as the complex physiological and psychological experience an individual undergoes when exposed to stimuli such as food (58). The most significant benefits of food typically stem from emotional factors tied to product intrinsic attributes, including sensory characteristics and preferences (59). When benefits are associated with risks, people tend to perceive these benefits as attributes that offset potential negative consequences. Wild mushrooms are cherished for their delicious taste and celebrated as “purely natural foods,” earning widespread popularity among Chinese consumers (9). In Central and Eastern European countries, wild mushrooms are valued for their unique texture, distinct flavor, and nutritional profile as a protein source. Nutritionists even promote them as meat substitutes, making them a long-standing favorite (44). When food benefits align with sensory appeal, environmental factors, or emotional associations, these perceptions often arise from individual product experiences (57, 60). When asked about the most important attributes influencing food choices, consumers most frequently cite taste (59). Consuming wild mushrooms offers the sensory pleasure of their unique flavor but also carries the risk of accidental poisoning. Food choices are primarily driven by preferences and situational context—what is most enjoyable and suitable for a given occasion—rather than risk assessment, which is often deprioritized by consumers (61).

In Chinese culture, wild mushrooms are not only prized for their distinctive flavor but also revered as “mountain delicacies” symbolizing high nutritional value (44). This cultural perception amplifies consumers' perceived benefit of wild mushrooms, elevating their prominence in food choices. Consumers also exhibit a preference for “pure natural” foods, associating them with minimal processing, traditional production methods, and desirable sensory qualities while expecting greater health benefits (59). Such perceived benefit directly influences individual consumption intentions.

Self-efficacy, a core concept proposed by American psychologist Albert Bandura in his social cognitive theory during the 1970s (62), refers to an individual's belief in their capability to execute tasks successfully. According to social cognitive theory, individuals with higher self-efficacy are more likely to believe they possess the ability to accomplish challenging tasks (63). Self-efficacy involves an assessment of one's capacity to perform tasks and represents a perceived capability. In the context of food safety, individuals with higher self-efficacy perceive themselves as having sufficient knowledge and confidence to avoid risks (28). Higher levels of self-efficacy are associated with lower perceived susceptibility to health risks such as avian influenza infection, thereby reducing the likelihood of taking preventive actions (64).

A study revealed that in some rural areas of Changsha, a city of China, residents have long maintained the dietary practice of foraging and consuming wild mushrooms. Older individuals, in particular, frequently disregard warnings from staff and continue harvesting and eating wild mushrooms as they strongly believe in their ability to identify toxic species based on personal experience (65). In regions of China where wild mushroom consumption is deeply rooted in cultural traditions, long-term consumption of specific species has fostered confidence in risk assessment. This confidence stems not only from personal experience but is also reinforced through inter-generational and community-based knowledge transmission (11). Such cultural and familial transmission of expertise strengthens consumers' self-efficacy, making them more inclined to consume wild mushrooms despite poisoning risks. Thus, in the Chinese cultural context, self-efficacy reflects not only individual capability but also the collective experience and knowledge inherited through family and community traditions.

Based on the above discussion, this study proposes the following hypotheses:

H3: Perceived benefit positively affects the willingness to eat wild mushrooms.
H4: Self-efficacy positively affects the willingness to eat wild mushrooms.

### 2.3 Demographic control variable

The propaganda degree of poisonous mushrooms varies from province to province, and the number of people who eat wild mushrooms and the poisoning rate will be different (66). In terms of gender differences, Zhitao et al. (67) found that the risk of male wild mushroom poisoning is higher than that of females, because men spend more time outdoors, prefer to collect wild mushrooms, and eat more wild mushrooms at the same time. In terms of occupational differences, Kalashnikov et al. found that in Ukraine, the occupations with a higher risk of mushroom poisoning are workers, unemployed people, and school students (68). In terms of age difference, Xun et al. (69) found that among patients with wild mushroom poisoning, the age group of 20–59 years old had the largest number of cases. The highest mortality rate is in the age group of 1–6 years old, followed by the age group over 60 years old. In addition, Rahayu et al. (70) found that a higher educational level may reduce the risk of poisoning by wild poisonous mushrooms. To sum up, our research takes province, gender, age, occupation, and education level as control variables.

**Figure 1:**
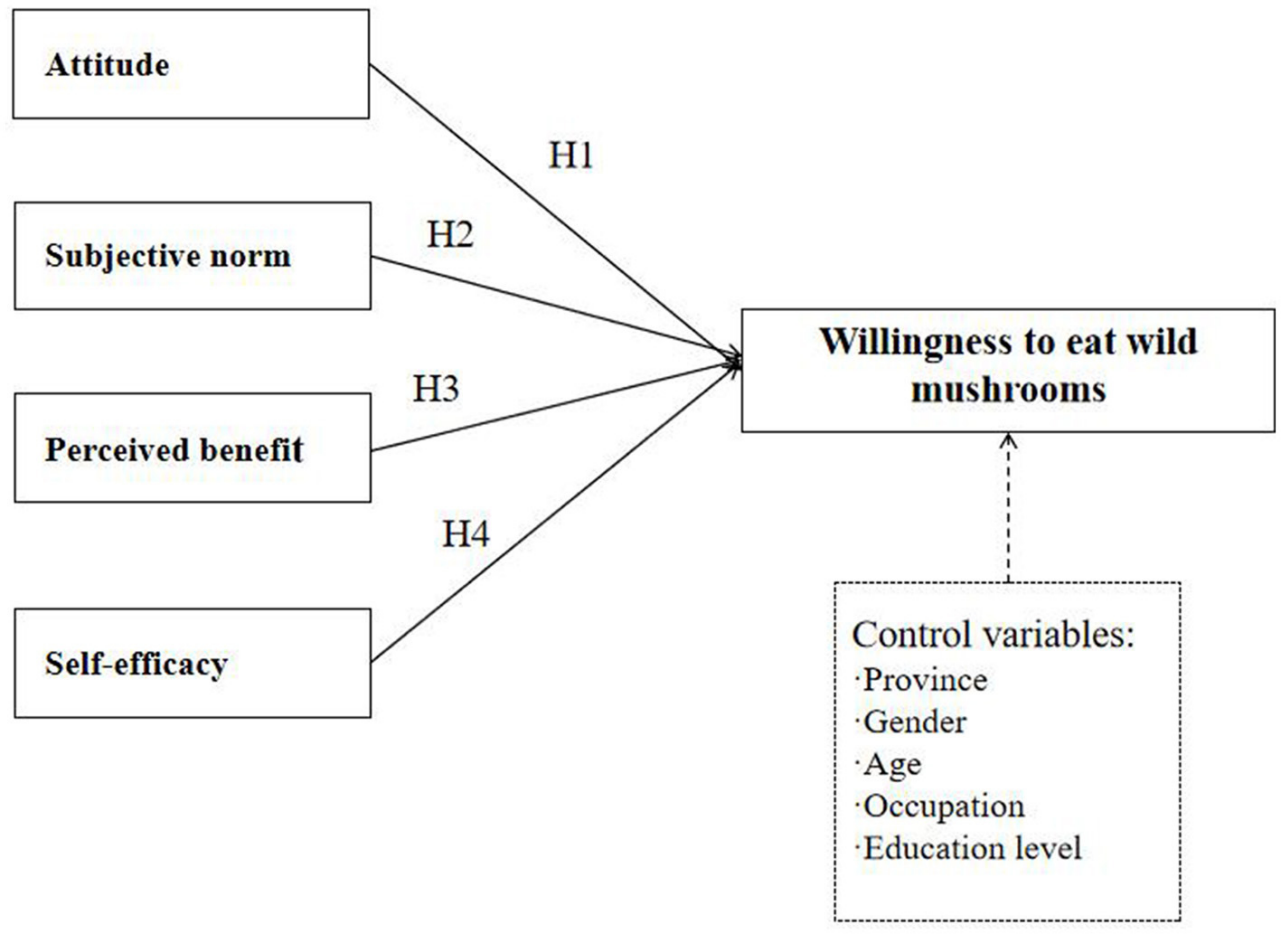
Conceptual framework.

Dietary behavior originates from a reasonable decision-making process and is influenced by many factors. Although the theory of reasoned action (TRA) primarily focuses on the two core factors of attitude and subjective norms, this study further extends the model by incorporating two additional dimensions: perceived benefit and self-efficacy, to more comprehensively understand consumers' willingness to eat wild mushrooms, as illustrated in Figure 1.

## 3 Methodology

### 3.1 Collection of research data

This study was designed and organized by researchers cooperating with China National Food Safety Risk Assessment Center, and data were collected in Guizhou, Hunan, Jiangxi, Yunnan, and Chongqing of China from 20 May 2021 to January 2022. In these areas, wild mushroom poisoning has been reported as the leading cause of death of food-borne diseases in the past few decades. Research data were collected through a combined approach of online surveys and in-person household surveys. The questionnaire design employed a five-point Likert scale (1: Strongly Disagree to 5: Strongly Agree). In the first section of the questionnaire, questions on age, gender, education, and income status were included to gather demographic variables (92). The second section addressed variables related to self-efficacy, perceived benefit, and constructs from the theory of reasoned action (TRA)—specifically, attitude, subjective norms, and intention to consume wild mushrooms. This study adopted a cross-sectional research design, characterized by collecting data from a population or specific group at a single point in time. The cross-sectional approach was chosen because it allows for a comprehensive understanding of the phenomenon under study while effectively assessing variations and relationships within the target population. After filling in the missing data by the mean interpolation method, 793 valid observations were finally included. Kline recommends a minimum sample size of 200 participants for structural equation modeling (SEM) (71). The study's sample size of 793 participants meets the required threshold for conducting structural equation analysis.

### 3.2 Research population and sample

As can be seen from Table 1, among the samples studied, Guizhou Province accounted for 16.3%, Hunan Province accounted for 12.4%, Jiangxi Province accounted for 16.9%, Yunnan Province accounted for 30.3%, and Chongqing City accounted for 24.2%. Most of them are young and middle-aged, accounting for 23.6% at the age of 18–40, 60.4% at the age of 41–60, 15% at the age of 61–80, and 1% over 81. In terms of gender distribution, women account for 46.2% and men account for 53.8%; in terms of educational background, 38.3% graduated from primary school, 34% from middle school, 15.8% from high school, 7.2% from university, and 4.6% from postgraduate. As far as occupations are concerned, 0.9% are students, 16.9% are homeworkers, 3.3% are unemployed, 4.8% are retired, 1.5% are government workers, 10.2% are technicians, 3.5% are office staff and related personnel, 8.2% are business workers, 39.3% are agriculture, forestry, animal husbandry, and sideline fishing, and 1.3% are farmers.

**Table 1:** Demographic variable statistics of the subjects (*N* = 793).

**Demographic indicators**	**Category**	**Population**	**Percentage**
Province	Guizhou Province	129	16.3%
	Hunan province	98	12.4%
	Jiangxi province	134	16.9%
	Yunnan Province	240	30.3%
	Chongqing	192	24.2%
Gender	Male	427	53.8%
	Female	366	46.2%
Age	18–40	187	23.6%
	41–60	479	60.4%
	61–80	119	15%
	≥81	8	1%
Educational level	Primary school	304	38.3%
	Junior school	270	34%
	Senior high school	125	15.8%
	University	57	7.2%
	Postgraduate	37	4.7%
Occupation	Students	7	0.9%
	Homeworker	134	16.9%
	Unemployment	26	3.3%
	Retired personnel	38	4.8%
	Government workers	12	1.5%
	Technicians	81	10.2%
	Office staff and related personnel	28	3.5%
	Business and service personnel	65	8.2%
	Production personnel of agriculture, forestry, animal husbandry, fishery, and water conservancy	312	39.3%
	Transportation personnel	13	1.6%
	Others	77	9.7%

### 3.3 Conceptual model and scale development

The conceptual model comprises five constructs, each measured through validated scales adapted from existing literature: attitude: adopted from Dean et al. (72), this scale consists of four items measuring an individual's emotional evaluation of consuming wild mushrooms. Subjective Norms: adapted from Menozzi et al. (73), this construct includes two items to assess perceived social pressure influencing behavior. Perceived benefit: adapted from Loh and Hassan (74), this scale includes three items evaluating the perceived advantages of consuming wild mushrooms. Self-Efficacy: derived from de Menezes et al. (75), this construct comprises two items reflecting confidence in one's ability to safely eat wild mushrooms. Behavioral Intention (Dependent Variable): willingness to eat wild mushrooms, the study's outcome variable, is measured by one item. The design of the conceptual model is illustrated in Figure 1, integrating these constructs to examine their hypothesized relationships and predictive pathways.

### 3.4 Data analysis

The study employed partial least squares structural equation modeling (PLS-SEM) using SmartPLS 3.0 to analyze the data. The choice of the PLS method was driven by its dual strengths in supporting both exploratory and confirmatory research (76), effectively handling complex interactions among multiple predictor variables (e.g., perceived benefit, self-efficacy, and other newly introduced constructs) while being suitable for moderate sample sizes (*n* = 793) (77).

First, the measurement model was validated through composite reliability (CR), Cronbach's α, and average variance extracted (AVE) to ensure internal consistency and convergent validity. Confirmatory factor analysis (CFA) was further conducted to evaluate discriminant validity among constructs. Subsequently, regression analysis was applied to examine the hypothesized path relationships between variables. Overall model fit indices (e.g., *R*^2^, effect size, and predictive relevance *Q*^2^) were used to assess the model's explanatory and predictive capabilities. This methodological approach balances the exploratory nature of extending the theory of reasoned action (TRA) with the rigor required for hypothesis validation, ensuring both theoretical innovation and empirical robustness.

## 4 Result

### 4.1 Measurement model results

With the SmartPLS 3.0 software, the measurement values of the model were obtained. The main statistical indicators showed satisfactory values exceeding reference benchmarks. Hair et al. (78) pointed out that when the number of measurement indicators for a variable is < 6, a Cronbach's α coefficient >0.6 indicates that the scale is reliable. The minimum Cronbach's α value was 0.683, exceeding the 0.6 threshold, indicating the reliability of the scale; convergent validity reflects the degree of aggregation of latent variables corresponding to observed variables. Convergent validity is primarily measured through factor loadings, composite reliability (CR), and average variance extracted (AVE) (79). Among these, factor loadings must be >0.60, composite reliability must exceed 0.80, and average variance extracted must be >0.50 (80). For all latent variables, the minimum factor loading value was 0.811, all exceeding 0.6, with CR above 0.859 and AVE values above 0.721, indicating that the scale's internal consistency and fit are sufficient (Table 2).

**Table 2:** Result of convergent validity and internal consistency reliability.

**Latent variables**	**Items**	**Item description**	**Factor loadings**	**CR**	**AVE**	**Cronbach's α**
Perceived benefit	V1	Wild mushrooms are delicious.	0.915	0.909	0.768	0.849
	V2	Wild mushrooms are very nutritious.	0.851			
	V3	Wild mushrooms are natural.	0.862			
Self-efficacy	V4	I am confident to avoid the risks posed by poisonous wild mushrooms.	0.9	0.906	0.828	0.793
	V5	I have enough knowledge to avoid the risk of poisonous wild mushrooms.	0.92			
Subjective norm	V6	Friends and relatives will want me to eat wild mushrooms with them.	0.917	0.859	0.753	0.683
	V7	The attitude of relatives and friends to wild mushrooms will affect whether I eat wild mushrooms (for example, if relatives and friends like to eat them, I will want to try them)	0.816			
Attitude	V8	Eating wild mushrooms will make my friends and family very happy.	0.827	0.912	0.721	0.872
	V9	Eating wild mushrooms will bring many benefits to my relatives and friends.	0.811			
	V10	Generally speaking, wild mushrooms are good food.	0.876			
	V11	On the whole, I am satisfied with wild mushrooms.	0.881			

The discriminant validity of our model was measured using two well-known methods, such as the Fornell–Larcker criterion and the heterotrait-monotrait (HTMT) ratio (81). The Fornell–Larcker criterion measures discriminant validity by taking the square root of the AVE values for all constructs (82, 83). Table 3 presents the Fornell–Larcker values constructed in this study. According to the threshold, all upper values in the table's columns should exceed the lower values. The results of this study meet the threshold specified by Fornell–Larcker because the upper values in Table 3, displayed in bold, exceed the values below them. Therefore, discriminant validity is confirmed in this study's model. In addition, according to the standard, the HTMT values for all constructs should ideally be < 0.85 and must be < 0.9 (81). As shown in Table 4, the HTMT values in our model are < 0.9. Therefore, the HTMT discriminant validity of this study's model is also achieved. In summary, the questionnaire is considered to have good discriminant validity.

**Table 3:** Result of discriminant validity.

**Construct**	**Attitude**	**Perceived benefit**	**Subjective norm**	**Self-efficacy**	**Willingness to eat wild mushrooms**
Attitude	**0.849**				
Perceived benefit	0.726	**0.877**			
Subjective norm	0.269	0.163	**0.91**		
Self-efficacy	0.679	0.436	0.156	**0.868**	
Willingness to eat wild mushrooms	0.741	0.602	0.317	0.607	1

The bold values of diagonal is the square root of AVE.

**Table 4:** Discriminant validity (HTMT).

**Construct**	**Subjective norm**	**Attitude**	**Perceived benefit**	**Self-efficacy**	**Willingness to eat wild mushrooms**
Subjective norm					
Attitude	0.855				
Perceived benefit	0.561	0.845			
Self-efficacy	0.201	0.318	0.198		
Willingness to eat wild mushrooms	0.715	0.781	0.652	0.355	

### 4.2 Structural modeling

The *R*^2^ values of the latent constructs explain the strength of the model, with values >0.5 indicating substantial strength. Our model's *R*^2^ is 0.601, demonstrating a large explanatory power. A *Q*^2^ value greater than zero is considered suitable for the model. The *Q*^2^ value of the latent constructs in this study's model is 0.591, exceeding zero, indicating that the tested model has predictive relevance. In addition, Table 5 also shows the variance inflation factor (VIF) values for all constructs in this study's model. VIF was examined to identify collinearity issues in the model. According to the standard, values below 5 are considered appropriate as they indicate no collinearity. Among the constructs in this study's model, the “attitude” construct exhibited the highest VIF value (3.396) compared to other items. Therefore, the results indicate that no collinearity issues exist in this study's model. Thus, all numerical values in this study meet the established criteria, confirming the meaningfulness of the model (81).

**Table 5:** Result of inner-model analysis.

**Item**	**R-squared (*R*^2^)**	**Adj. *R*^2^**	**Effect size (*f*^2^)**	***Q*^2^ predictive validity**	**VIF**
Attitude			0.136		3.396
Perceived benefit			0.037		2.164
Subjective norm			0.064		1.888
Self-efficacy			0.048		1.095
Willingness to eat wild mushrooms	0.606	0.601		0.591	

**Figure 2:**
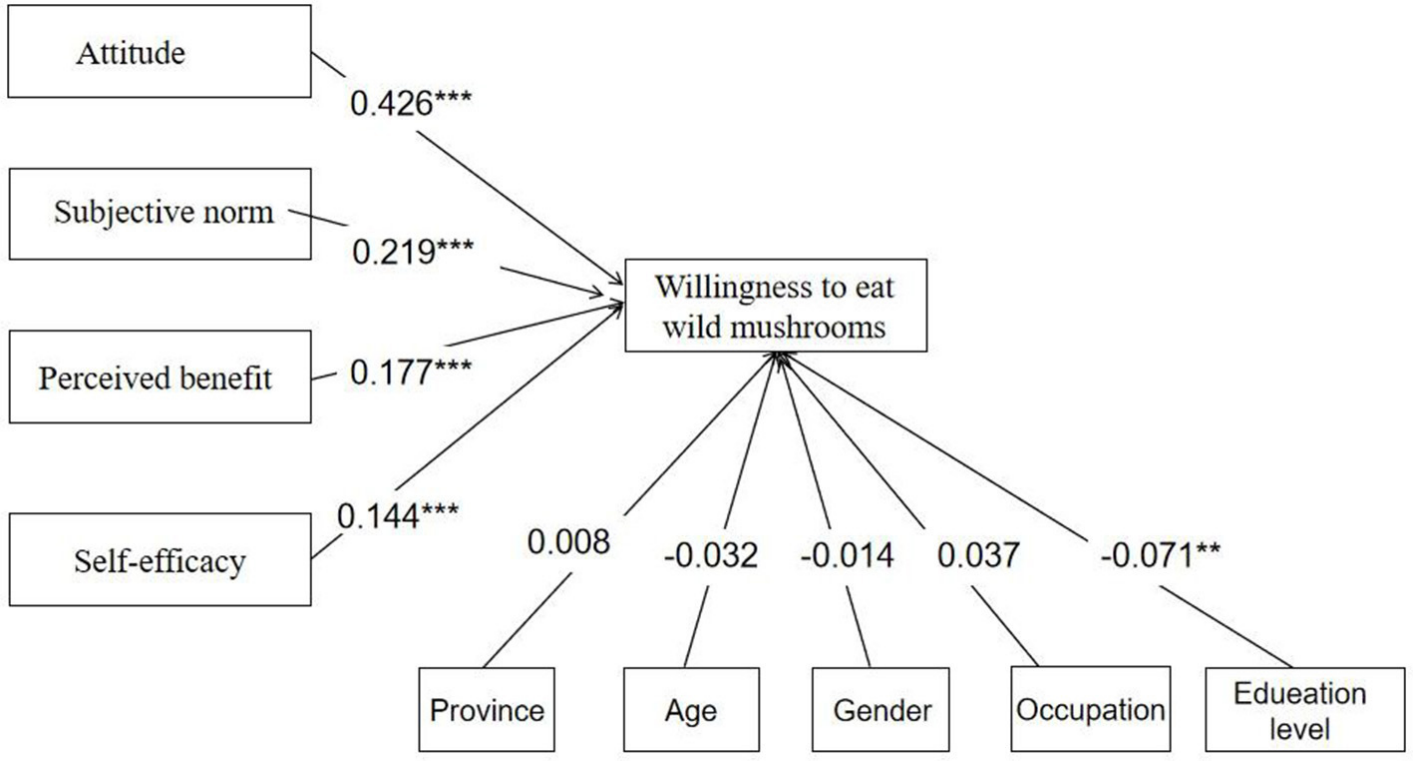
Statistically tested study model. ^**^p < 0.01; ^***^p < 0.01.

Furthermore, among the control variables (province, gender, age, occupation, and education level), only education level had a significant impact on the willingness to eat wild mushrooms. Education level has a negative effect on residents' willingness to eat wild mushrooms; the lower the education level, the higher the willingness to eat wild mushrooms (Figure 2).

To ensure that these positive relationships were truly statistically significant, we applied a bootstrapping technique (2,000 subsamples). Here, we find that all such values were significant, as shown in Table 6.

**Table 6:** Hypothesis testing for relationships among variables.

**Relationship**	**Original sample**	**Sample mean**	**Standard error**	***T* statistics**	***P***	**Decision**
Attitude → willingness to eat wild mushrooms	0.426	0.427	0.052	8.237	*P* < 0. 001	Accept
Subjective norms → willingness to eat wild mushrooms	0.219	0.221	0.041	5.314	*P* < 0.001	Accept
Perceived benefit → willingness to eat wild mushrooms	0.177	0.174	0.039	4.586	*P* < 0.001	Accept
Self-efficacy → willingness to eat wild mushrooms	0.144	0.144	0.024	5.905	*P* < 0. 001	Accept

## 5 Discussion

The primary objective of this study is to investigate the impact of behavioral predictors on consumers' willingness to eat wild mushrooms, proposing an extended theory of reasoned action (TRA) model that incorporates two additional variables: perceived benefits and self-efficacy. In addition, the study considers the collectivist historical and cultural traditions within the Chinese sociocultural context, emphasizing the cultural and psychological characteristics underlying the behavior of consuming wild mushrooms.

First, the study results indicate that attitude has a positive effect on the behavioral intention to eat wild mushrooms, consistent with previous findings (84). Attitude is believed to influence individual behavior; individuals who believe a behavior will lead to positive outcomes are likely to have a positive behavioral intention, while negative attitudes may lead to unfavorable behaviors (93). Wild mushrooms are part of traditional dietary habits in some regions, and local residents often hold positive and affirmative attitudes toward them. Consumers' attitudes toward consuming wild mushrooms depend on their knowledge of mushrooms and awareness of poisoning risks. Currently, residents in regions with abundant wild mushrooms often lack adequate knowledge to identify toxic species, and their foraging practices are largely unregulated by authorities (85). Consumers may overestimate their food safety knowledge and fail to recognize that their understanding may not reflect actual risks (86). In future, wild mushroom safety education, marketing, and government agencies could emphasize the negative consequences of unsafe food practices through media campaigns to enhance consumers' awareness of the risks associated with toxic mushrooms, particularly discouraging the consumption of mushrooms they cannot confidently identify. Evans et al. (87) found that as awareness of food-borne illnesses deepens, risk-taking behaviors gradually decrease.

Second, this study further validated the direct influence of subjective norms on dietary behavior in societies that emphasize collective, a finding consistent with research by Soon et al. (88) and Kurniawan et al. (89) in Indonesia and Malaysia, which can be explained by the interdependent cultural values of these nations. China is a society that emphasizes collective where individuals belong to strong and cohesive groups or extended families, and other's opinions may hold greater weight. Normative expectations and responsibilities shape individual attitudes in societies. The Greek term for food, “oikos,” means “family” and originally referred to “a group that eats together” (90). In China, “the process of social cohesion is always inseparable from dining” (86). What to eat, where to eat, and other food-related decisions have become critical criteria for the reorganization of groups and social stratification. Thaivalappil et al. (91) proposed that others' expectations regarding how food is handled at home and the social responsibilities of being a cook may benefit educational initiatives. Ruby et al. (33) found that consumers are more likely to consider and follow the advice of those closest to them.

Third, this study found that perceived benefit has a positive effect on the consumption of wild mushrooms, consistent with previous findings (44). Specifically, participants who more strongly recognize the benefits of consuming wild mushrooms (such as nutritional value, taste experience, or local cultural significance) are more likely to exhibit higher consumption willingness. This finding underscores the importance of perceived benefit in influencing individuals' behavioral decision-making processes (94). These results suggest that educational campaigns and promotional materials should highlight the potential risks of wild mushrooms (e.g., poisoning risks) while emphasizing the importance of proper identification and handling. This approach encourages consumers to consider both the benefits and risks when deciding to eat wild mushrooms.

Finally, this study found that food self-efficacy has a positive effect on the consumption of wild mushrooms, consistent with previous literature. JM Abbot et al. found that younger individuals exhibit higher self-efficacy regarding food safety, believing they possess sufficient knowledge to avoid risks. In the context of consuming wild mushrooms, individuals who perceive themselves as capable of identifying, foraging, and cooking wild mushrooms, or who believe their access to wild mushrooms is safe and reliable, are more likely to develop consumption intentions. This is because self-efficacy influences individuals' confidence in performing a behavior; when individuals believe they can successfully execute a behavior, they are more likely to engage in it.

Rural residents, compared to urban residents, have greater exposure to and use of wild mushrooms. However, due to lower educational attainment among rural residents and the morphological similarity between some edible and toxic mushrooms, rural areas experience significantly more wild mushroom poisoning incidents than urban areas (85). This study also found that groups with lower educational levels are more likely to exhibit a willingness to eat wild mushrooms. These individuals often lack knowledge about preventing toxic mushroom poisoning. Recommendations include reorganizing scientific information to address high-risk audiences' misconceptions (e.g., “I have sufficient identification ability; since I was not poisoned before, I will not be poisoned in the future”), specifically highlighting locally confusing toxic mushroom species to challenge audiences' overconfidence in their identification skills and correct such misconceptions.

These research findings provide critical entry points for public health policies. In cultures that emphasize collective, individual behavior is more driven by social norms, i.e., the influence of “group pressure” (e.g., expectations from family and friends), on behavioral intentions is stronger. Policy design can draw inspiration from Indonesia's “Family Food Safety Ambassadors” program (88), recruiting rural doctors or community leaders to conduct regular home visits to train residents to analyze the information of poisonous mushrooms and share poisoning case videos via social media groups, thereby embedding risk education into daily social interactions. Additionally, it is necessary to further standardize the wild mushroom trading through the identification of technical specifications, such as the policy practice of wild mushroom trading in Italy through molecular identification (10), thereby reducing risks at the source of the supply chain.

## 6 Conclusion

This study examines risk behaviors by emphasizing a very unique cultural background, in which people eat wild mushrooms as part of cultural habits, and in the past few decades, wild mushroom poisoning has been identified as a high mortality rate in some areas of China. To understand the predictive factors of willingness to take risks, we verified several important factors, such as perceived benefit and food self-efficacy, and we also tested the influence of subjective norms and attitudes on behavioral willingness. Our research contributes to the current understanding of risk cognition and related behaviors and extends to the discussion of social and cultural background characteristics and collective cultural values. We call for further research to test the risk perception and communication paradigm, transcend the cultural tradition centered on the Western paradigm, and develop a cross-cultural perspective that emphasizes the uniqueness of the local cultural background.

## 7 Limitations and future research

This study has several limitations. First, the sample's over-representation of rural participants from high-incidence southwestern China may have overestimated population-level risk preferences, necessitating future inclusion of urban-eastern populations to enhance generalizability. Second, self-reported data may have been influenced by social desirability bias, warranting validation through behavioral records or experimental methods. Third, unmeasured environmental and economic factors (e.g., foraging accessibility and household income) could moderate outcomes, requiring inclusion in future models. Finally, the theoretical framework's focus on collectivist cultural contexts limits its applicability to individualistic societies, which remains to be tested.

Future studies can deepen the following directions: first, conduct cross-cultural comparisons to validate the model's applicability in individualistic societies, particularly focusing on differences in the role of subjective norms and exploring how cultural dimensions (e.g., uncertainty avoidance) moderate risk decision-making mechanisms. Second, adopt a mixed-methods design: use longitudinal tracking to reveal seasonal variations in risk perception between rainy and dry seasons, and design randomized controlled experiments (e.g., comparing the effects of household advocate training vs. community poster interventions) to quantify the efficacy of policy interventions. Third, integrate social media big data to capture unconscious consumer reactions to risk information and analyze patterns of public opinion dissemination. Fourth, expand the theoretical framework by integrating the health belief model (HBM) and theory of reasoned action (TRA), incorporating moderating variables such as economic dependency, to construct a multidimensional predictive model. Finally, focus on high-risk groups (e.g., older foragers and migrant workers) through participatory research to develop customized intervention tools, and establish a dynamic policy evaluation system to achieve targeted resource allocation.

## Data Availability

The original contributions presented in the study are included in the article/supplementary material, further inquiries can be directed to the corresponding author.
